# Precise Measurement of Gas Volumes by Means of Low-Offset MEMS Flow Sensors with μL/min Resolution

**DOI:** 10.3390/s17112497

**Published:** 2017-10-31

**Authors:** Massimo Piotto, Simone Del Cesta, Paolo Bruschi

**Affiliations:** 1 IEIIT-PISA, CNR, Via G. Caruso 16, 56122 Pisa, Italy; massimo.piotto@ieiit.cnr.it; 2 Department of Ingegneria dell’Informazione, University of Pisa, 56122 Pisa, Italy; simone.delcesta@ing.unipi.it

**Keywords:** gas volume measurement, MEMS flow sensor, thermal flow sensor, system on a chip

## Abstract

Experiments devoted to evaluate the performance of a MEMS thermal flow sensor in measuring gas volumes are described. The sensor is a single-chip platform, including several sensing structures and a low-offset, low-noise readout interface. A recently proposed offset compensation approach is implemented obtaining low temperature drift and excellent long time stability. The sensor is fabricated by applying a simple micromachining procedure to a chip produced using the BCD6s process of STMicroelectronics. Application of a gas conveyor allowed inclusion of the sensing structure into a channel of sub-millimeter cross-section. The results of measurements performed by making controlled air volumes pass through the sensor channel in both directions at rates from 0.1 to 5 mL/min are described.

## 1. Introduction

Fine measurements of gas volumes through pipes or ducts are required in many application fields, including natural gas metering, patient respiration monitoring and drug delivery. A very simple method to measure the gas volume is the integration over time of the output of flow rate sensors. However, this method poses tight boundaries on the characteristics of the flow sensor in order to obtain a precise measurement. Due to the necessity to integrate the sensor data, offset components produce an error that grows with time. For this reason, in order to enable volume measurements over long periods, flow sensors should be marked by very low offset and offset drift. Devices should also have fast response times in order to cope with impulsive or highly irregular flows. Furthermore, low power consumption is an additional requirement for battery-powered instruments or for applications, like natural gas metering, where there is a large distribution network that requires a great number of devices. Due to their typically slow response and high power consumption, macroscopic thermal flow sensors [1] have been rarely used in volume metering applications, despite their useful characteristics like absence of moving parts, high sensitivity and wide dynamic range. Nowadays, MEMS technologies allow fabrication of thermal flow sensors with response times in the order of milliseconds and power consumptions of only a few milliwatts [2,3,4,5]. Furthermore, these sensing structures could be integrated on the same chip with the read-out circuits, obtaining very compact devices with high immunity to electromagnetic interference [6,7,8,9,10]. For these reasons, MEMS thermal flow sensors have started to be proposed for natural gas metering applications [11] and they are considered a promising substitute of the conventional diaphragm meters [12]. Integrated thermal flow sensors have been proposed for the fabrication of portable spirometers [13,14] as an effective alternative to capacitive [15,16] and turbine-type sensors [17].

In this work, we describe a series of experiments aimed at demonstrating the possibility of using a recently proposed flow sensor [18] for precise measurements of air volumes. The sensor is based on a silicon chip including several flow-sensitive microstructures and a high-precision readout interface. The sensing structures convert the flow rate into a temperature difference exploiting the differential heat transfer mechanism [19]. The temperature difference is then converted into a voltage by means of micro-thermopiles. The chip is fabricated using the Bipolar-CMOS-DMOS process BCD6s of STMicroelectronics, followed by ad hoc post-processing steps aimed at suspending the sensor elements and achieving the required thermal insulation. Very low offset equivalent flow is obtained by reading the thermopile voltage with a chopper amplifier and adopting a recently proposed [20] low-drift compensation procedure to cancel the offset contributions from the sensing structure. 

Experimental data presented in this paper include characterization of the time and temperature stability of the output offset, due to the importance of this parameter for volume metering applications. The sensor capability of measuring very small air volumes delivered at different flow rates is thoroughly investigated using an accurate syringe pump.

## 2. Description of the Sensor Architecture and Fabrication

### 2.1. System Architecture

The flow sensor is based on a silicon chip that includes both the sensing structures and a readout interface (SoC: System on a Chip). The chip has been designed using a standard microelectronic process (BCD6s of STMicroelectronics, Milan, Italy) followed by a post-processing procedure, required to selectively remove the silicon substrate under the elements of the sensing structures. In this way, thermally insulated membranes, suspended over cavities etched into the substrate, are created. Details on the post-processing technique are described in Section 2.2. 

**Figure 1:**
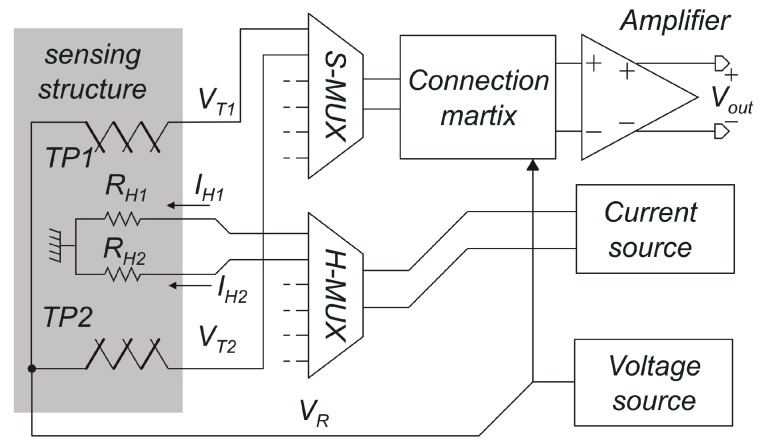
Simplified architecture of the sensor System on a Chip (SoC). The amplifier gain is 200. The digital subset is not shown for simplicity.

A simplified diagram of the SoC is shown in Figure 1. All electronic blocks are controlled by digital codes that can be set by means of an SPI (Serial Peripheral Interface) communication line in order to implement different functions. The digital subset is not shown in Figure 1 for simplicity. The sensing structure is based on the differential heat-transfer principle [19] and is formed by two resistive heaters (p-polysilicon resistors, *R_H1_* and *R_H2_* in the figure, 2 kΩ each) placed between two thermopiles formed by ten p-poly/n-poly thermocouples (*T_P1_* and *T_P2_*). The total thermopile sensitivity is 4 mV/K. All the four elements are suspended on independent silicon dioxide membranes, so that heat transfer from the heaters to the thermopiles occurs mainly through the gas.

The heaters are driven by two independent currents, *I_H1_* and *I_H2_*. The thermopiles hot junctions face the heaters, whereas the cold ones lie on the substrate, so that the thermopile voltages (*V_T1_* and *V_T2_*) are proportional to the temperature difference between the hot junctions and the substrate. The gas flows in the direction that crosses the four sensor elements and, due to forced convection, shifts the heat transfer downwind. In this way, the downwind thermopile measures a higher temperature with respect to the upwind one and a differential temperature *V_T1_*–*V_T2_* is produced. The sensing structure is connected to the read-out electronics by means of two analog multiplexers that allow selection of one of the three different sensing structures present on the chip. The thermopile voltages *V_T1_* and *V_T2_* are fed to a switch matrix that, depending on a digital control code, feeds the amplifier inputs with different signals. The four possibilities are shown in Table 1.

Flow measurements are performed programming the switch matrix with code =3, so that the amplifier processes the differential voltage *V_T1_*–*V_T2_*. Acquisition of the individual thermopile voltages (codes 0 and 2) is used for characterization and diagnostic purposes as well as for implementation of simultaneous flow and pressure measurements according to the algorithms described in [21]. All the tests described below are performed with code =3. The possibility to short circuit the amplifier inputs is used to estimate the amplifier contribution to the total offset. 

The double-heater configuration is used to implement the sensor-offset cancellation approach described in [10,20]. The sensor offset, i.e., the voltage produced by a sensing structure in zero-flow conditions, is due to unavoidable asymmetries of the structure. This problem affects also single heater structures and occurs when, for example, the heater is slightly closer to one of the two thermopiles. With the double-heater structure, the offset is compensated by applying a differential component to the two currents that are fed to the heaters. The programmable current source included into the chip is capable to fine-tune the differential component, which is controlled by a 10-bit code. In this way, differently from other conventional offset compensation approaches, the sensor offset drift is also significantly reduced [20]. Note that noise superimposed on the heater currents results in temperature fluctuations that may give an important contribution to the thermopile noise voltage. For this reason, the current source has been designed taking into account strict constraints on the total noise magnitude. In particular, large-area MOSFETs have been used to reduce flicker noise, which is the dominant component at low frequencies. 

The amplifier uses an indirect current feedback (ICF) [22] architecture, accomplishing chopper modulation to obtain an input offset voltage smaller than 2 μV. Synchronous port swapping is implemented to improve gain accuracy and boost the input resistance. Details on the amplifier topology and performances are given in [23]. The amplifier gain is set to 200, while the bandwidth is reduced to 200 Hz in order to shape the output noise and reject chopper artifacts (offset ripple). Voltage *V_R_* (1.4 V) provides the optimal common mode voltage to the amplifier inputs. The whole chip operates at 3.3 V supply voltage with 2.6 mW total power consumption. 

**Figure 2:**
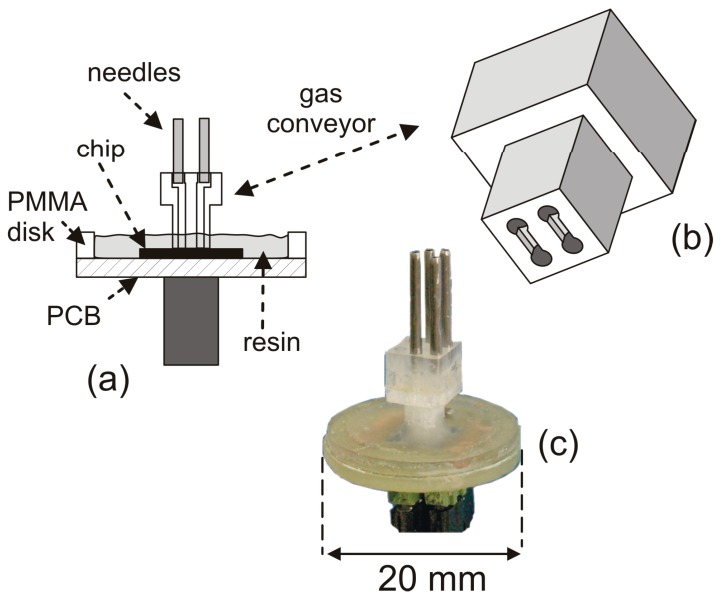
(**a**) Schematic view of the sensor after packaging; (**b**) perspective view of the gas conveyor showing the trenches milled onto the face that is applied to the chip; (**c**) photograph of the finished sensor.

The structure of the finished sensor is shown in Figure 2a. The chip is glued to a circular Printed Circuit Board including copper tracks for providing the required contacts and a single power-supply bypass capacitor (1 μF). Connection between the chip bonding pads and the copper tracks was performed by means of wedge bonding. A poly(methyl-methacrylate) (PMMA) gas conveyor, shown in the perspective view of Figure 2b, is applied to the chip surface in order to include the sensing structures in proper flow channels. The latter are obtained by milling trenches on the conveyor face that is applied to the chip. The conveyor is aligned to the chip in such a way that each sensing structure of interest falls into the centerline of a single trench. The trenches are sealed by the chip surface forming in this way channels that are accessed from the opposite face of the conveyor through holes drilled at the ends of the trenches. A layer of fluid silicone-based glue is deposited on the conveyor face prior to placing it in contact with the chip, in order to improve sealing of the flow channels. Alignment of the conveyor to the chip is performed manually, by means of a four-axis (*x*,*y*,*z*,*θ*) micromanipulator and a low magnification microscope. The flow channel cross-section is rectangular, with 0.5 mm × 0.15 mm (width × height) dimensions. Cyanoacrylate resin is poured around the chip to provide mechanical robustness. Using this procedure, it is possible to convey multiple gas flows to the chip [24], allowing fabrication of single-chip, multiple port flow sensors. The sensor proposed in this work is equipped with a two-port gas conveyor, whereas only one channel is actually used in the experiments. A photograph of the finished device is shown in Figure 2c.

### 2.2. Fabrication Process

**Figure 3:**
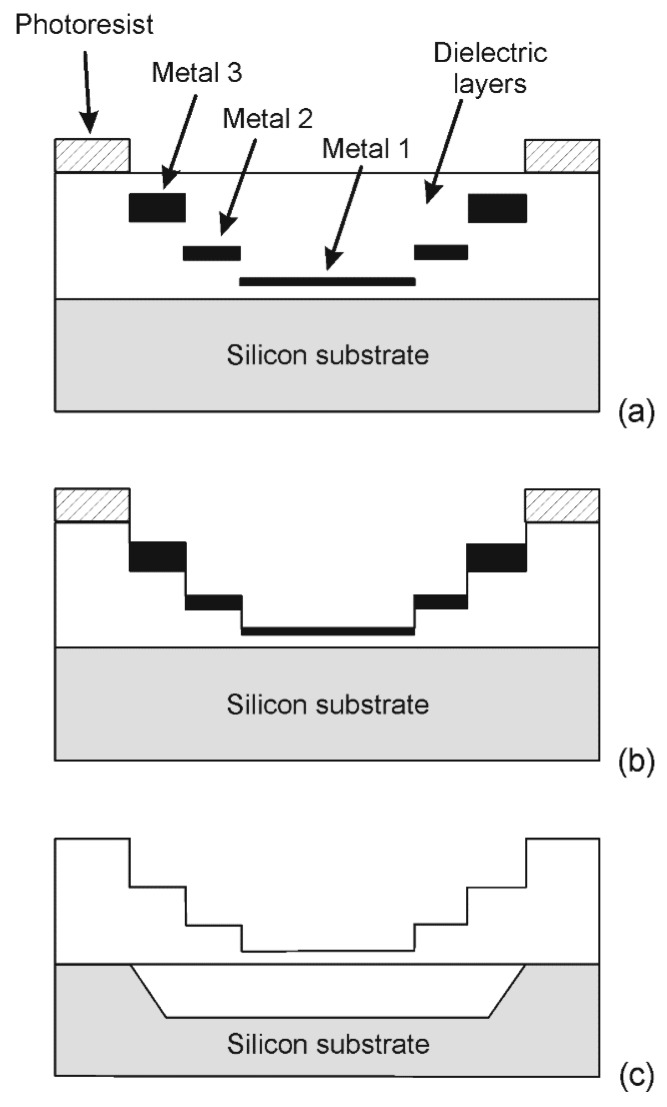
Schematic view of the post-processing procedure (not to scale): (**a**) definition of a thick photoresist layer; (**b**) etching of the SiO_2_ with a fluorocarbon-based plasma; (**c**) removal of the masks and anisotropic etching of the silicon substrate through openings out of the represented cross-section.

The chip has been designed with the BCD6s process of STMicroelectronics and a simple post-processing procedure has been applied in order to obtain suspended structures, which are thus thermally insulated from the substrate. The post-processing is aimed at selectively removing the dielectric layers from the front side of the chip and then opening a cavity in the substrate by means of anisotropic silicon etching. The dielectric layer removal is based on a novel technique that allows modulating the thickness of the suspended structures without adding lithographic steps. The technique, which represents an evolution of the techniques described in [25], exploits the selectivity toward aluminum of fluorocarbon-based plasma etchings of SiO_2_. The metal layers of the IC (Integrated Circuit) fabrication process can be used to mask the etching locally, as schematically shown in Figure 3 for a three metal level process. The photoresist is used to mask the rest of the chip and to define areas where the complete stack of the dielectric layers should be preserved. After the plasma etching, the metal masks can be removed with a wet etch using the same photoresist layer of the dry etching as a mask. As a result, the dielectric stack thickness can be quantized into (*N* + 1) different levels in a process with *N* metal layers, using a single lithographic step. This feature is useful for the fabrication of MEMS microstructures where parts of different thicknesses are often required. 

Tapered membranes could be fabricated with the proposed technique obtaining a good compromise between thermal insulation and mechanical robustness. Another advantage is that the metal masks are placed during the chip layout design with the resolution of the IC process. Thus, there is the possibility of defining the sensing structures with the metal masks and using the photoresist only to protect the rest of the chip. In this way, the resolution requirements of the post-processing procedure are significantly relaxed with clear advantages in terms of costs and yield. It is important to stress the advantage of exploiting layers (metal interconnects) that are already included in most standard microelectronic processes. Thus, non-standard micromachining procedures are kept to a minimum. 

**Figure 4:**
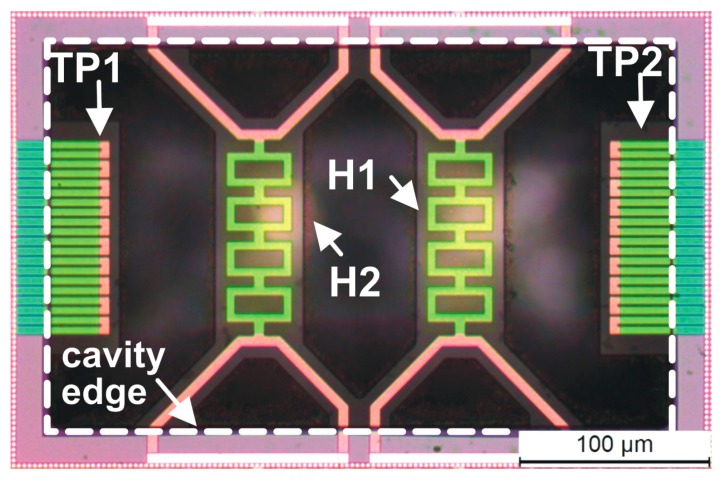
Optical micrograph of the sensing structure at the end of the micromachining procedure. Labels are as follows: TP1, TP2: thermopiles, H1, H2: heaters.

As far as the sensing structures proposed in this work are concerned, the heater membranes and the thermopile cantilever beams were defined using the Metal 2 layer, which represents a good trade-off between robustness and thermal insulation. Details about the post-processing procedure are reported in [18]. Briefly, a Reactive Ion Etching (RIE) in CF_4_/Ar (50%/50%) gas mixture with a RF power of 100 W was used to remove the dielectric layers using the Metal 2 layer to locally define the sensing structure geometry. An 8 μm thick photoresist film (MEGAPOSIT™ SPR™ 220-7.0) was used to broadly select the sensor area and protect the rest of the chip. After the RIE step, the metal mask was removed with a H_3_PO_4_–HNO_3_–CH_3_COOH water mixture at 45 °C using the photoresist layer as pad protection. Then photoresist was removed with acetone at room temperature and the silicon substrate was anisotropically etched for 150 min at 85 °C in a solution of 100 g of 5 wt % TMAH with 2.5 g of silicic acid and 0.7 g of ammonium persulfate. The sensing structure at the end of the post-processing is visible in the optical micrograph shown in Figure 4, where the main sensor elements are indicated.

## 3. Results and Discussion

### 3.1. Experimental Setup

The main experiments presented in this work were aimed at determining the possibly of measuring gas volumes that pass through the sensor by simple integration of the output voltage. Ambient air was injected into the sensor by means of a syringe pump. With this approach, it is possible to precisely control the injected volume and to alternate suction and injection phases. Another method allowing generation of very small flow rate and precise monitoring of the injected volumes is based on using a gravity-driven water flow to displace the desired airflow [26]. This second approach produces much smoother airflow than a syringe pump, but the flow is less controllable. We preferred to use a syringe pump since it can be easily programmed to generate very reproducible suction/injection cycles.

**Figure 5:**
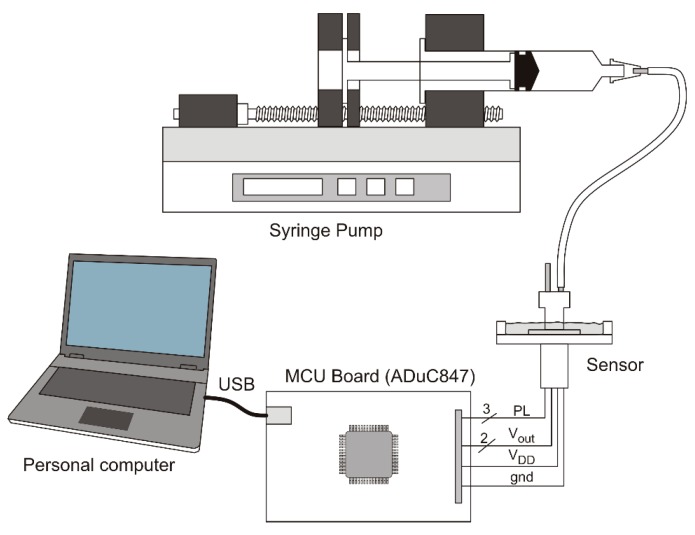
Measurement setup used for the volumetric measurements.

Air temperature was measured by means of a PT100 resistive probe. A simplified diagram of the experimental set-up is shown in Figure 5. The syringe pump was of reversible type, allowing air to be pumped or sucked into the sensor, depending on the direction of the stepping motor that controls the syringe piston. Syringes of two different diameters were used depending on the target volume. Connection between the sensor and the syringe is accomplished by means of a silicone pipe. The sensor is connected to a purposely-built printed circuit board, equipped with the ADuC847 micro-controller unit (MCU) of Analog Devices. The MCU controls the sensor settings through the serial program line (PL) and reads the analog voltages by means of the internal 24-bit ∆-∑ Analog-to-Digital converters (ADCs). The ADCs were programmed to provide a 40 sample/sec conversion rate. Due to the filtering properties of the ∆-∑ ADCs, the channel bandwidth was limited to 20 Hz, obtaining effective shaping of the output noise. The MCU board communicates with a personal computer via a USB interface. All the measurement process is controlled by a program running on the personal computer and written using the free Python multi-platform language. Numerical calculations are based on the Python “Numpy” module whereas the graphical user interface relies on the “PyQt4” porting of the “QT” C++ graphical libraries. 

The output voltage is converted into the corresponding mass-flow rate (sccm—standard cubic centimeters per minute) by means of cubic spline interpolation of a pre-loaded calibration table. Mass flow rate is converted into actual volume, taking into account the temperature and pressure of the ambient air. Volumes are simply calculated by means of real-time numerical integration of the instantaneous flow rate.

Characterization of the output-voltage vs. flow rate response was performed using a reference gas line equipped with precision mass flow controllers (MFCs). Two distinct MFCs, with full-scales of 10 and 200 sccm, were used to cover the whole flow range used for the sensor characterization. The input source of the gas line was a compressed air reservoir fed by an air compressor.

### 3.2. Sensor Calibration and Characterization

**Figure 6:**
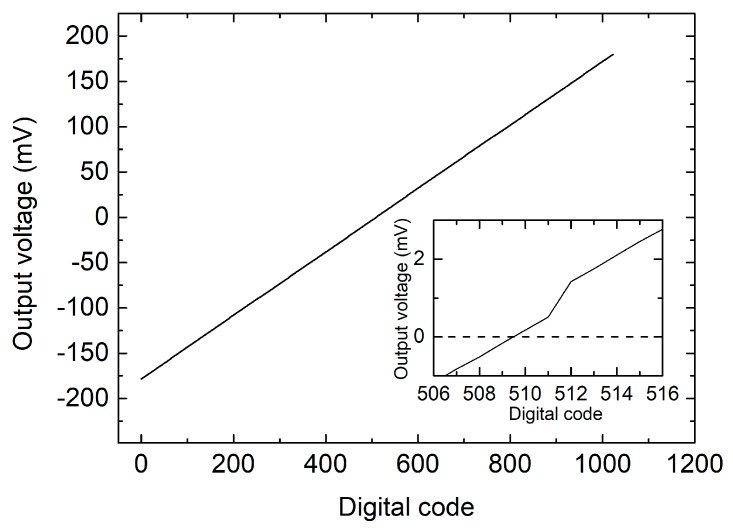
Output voltage as a function of the digital code that controls the differential component of the heater currents, properly trimmed to cancel the output offset.

**Figure 7:**
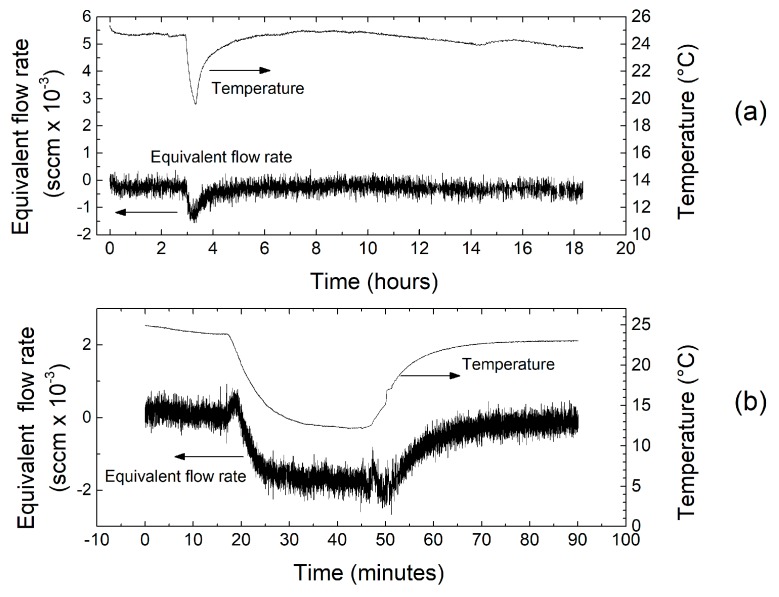
Recording of the output voltage, converted to an equivalent flow rate, across an 18 h interval (**a**) and a 90 min interval (**b**). During the shorter interval, a large temperature change is imposed.

Sensor calibration consisted of two distinct steps, namely (i) offset compensation and (ii) characterization of the sensor response. Offset compensation was obtained by scanning the 10-bit digital code that sets the differential component of the heater currents. This operation was operated by means of an automatic routine in zero flow condition. The result is shown in Figure 6, where the output voltage (i.e., the output offset) is plotted across the whole range of digital codes (0–1023). The midscale code (512) makes the two heater currents to be nominally equal. The inset shows that the output offset is minimized with codes 509 and 510. We chose the latter, obtaining a residual output offset of nearly 150 μV (0.75 μV referred to the amplifier input). The fact that the optimum code is very close to the midrange value means that the required correction term is very small. In particular, the relative current mismatch introduced to cancel the offset is only 0.08% of the nominal value. The residual part of the offset indicated above was cancelled by means of a simple subtraction in the digital domain. The long-term stability and temperature drift of the offset have been characterized by recording the sensor output voltage for several hours while keeping a zero flow-rate condition. Figure 7a shows the output voltage, converted into an equivalent flow rate, recorded over a period of 18 h. The figure shows also the evolution of the temperature during the measurement. The temperature drop at nearly 3 h from the start is due to unintentional opening of a window in the laboratory. The total integrated volume across the 18 h test was 0.332 mL, equivalent to a leakage of nearly 18.5 μL/h. The offset shows a good stability over time but some dependence on temperature is also visible. To further investigate this aspect, we have intentionally applied a larger temperature variation, obtained by moving the whole setup outside the laboratory after nearly 20 min from the beginning of the experiment, and then bringing it back into the lab after a 30 min exposure to outdoor air. In this way, a temperature variation of nearly 12 °C was applied. The result is shown in Figure 7b, where a total variation of nearly 2 × 10^−3^ sccm can be estimated, corresponding to a drift of slightly less than 2 × 10^−4^ sccm/°C. Repeating the same experiment while keeping the amplifier inputs short-circuited (code 1 in Table 1) did not result in any visible temperature drift. This fact clearly indicates that the offset drift originates from the sensor rather than the amplifier.

**Figure 8:**
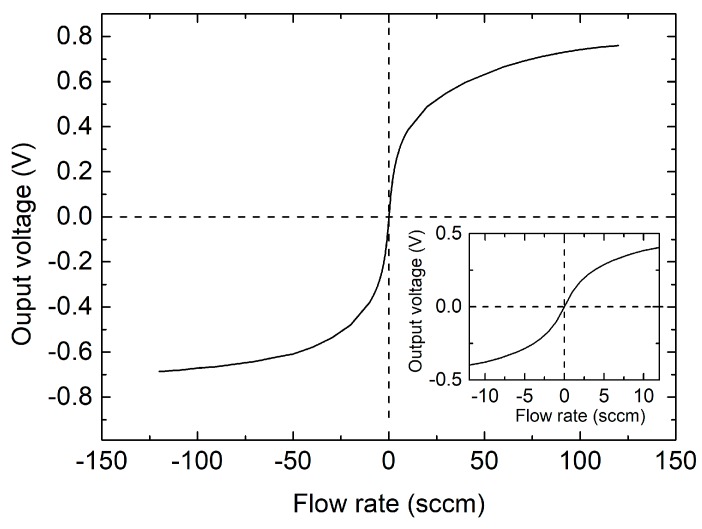
Output voltage vs. air flow-rate characteristic curve of the sensor. The inset shows a magnification at small flow rates.

**Figure 9:**
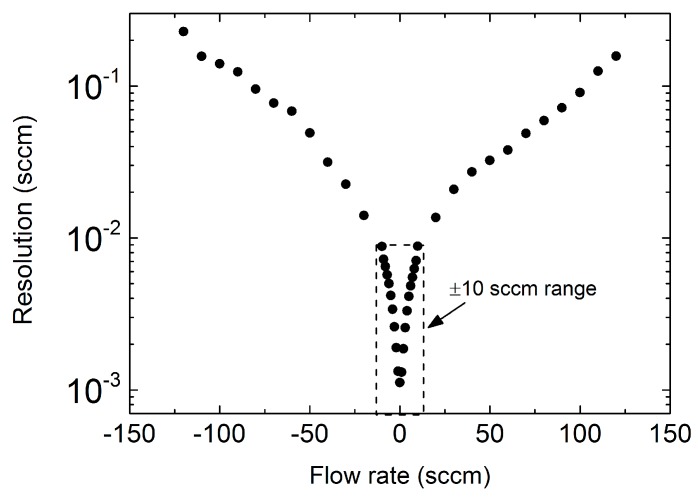
Sensor resolution as a function of flow rate. Resolution is reliably better than 10^−2^ sccm throughout the ±10 sccm interval, indicated by the dashed box.

After offset calibration, the output voltage vs. flow rate characteristic was measured over the 0.1–120 sccm range. The result is presented in Figure 8, where the inset shows a magnification of the curve for small flow rates. Negative flows are obtained by swapping the gas inlet and outlet ports of the sensor. The curve presents a strong non-linearity, with a tendency to saturate, typical of sensors based on differential heat transfer [19,27]. This behavior has a negative impact on resolution, which can be identified with the peak-to-peak equivalent noise flow-rate, *Q_pp_*, given by

(1) $$Q_{pp}=\frac{v_{n-pp}}{S}$$

where *v_n−pp_* is the peak-to-peak output voltage noise, while *S* is the sensitivity, defined as the derivative of the sensor characteristic. Resolution estimates have been obtained using an output noise level of 112 μV, peak-to-peak, estimated from the ADC output data stream in condition of zero flow. A sensitivity degradation of two orders of magnitude is apparent at high flow rates in the results shown in Figure 9. Note that a resolution better than 10^−2^ sccm is maintained for flow rates smaller than 10 sccm. All the volumetric experiments presented in next sub-section have been performed with flow rates in this restricted range.

Volumetric tests have been performed using the setup of Figure 5. In all tests, the syringe pump was programmed to suck a given air volume at a constant rate and then to inject the same volume back through the sensor at the same flow rate as in the suction phase. In the rest of this paper, the convention is that negative flows go from the sensor to the syringe (suction) while positive ones are injected by the syringe through the sensor. Volumes are negative since, due to the procedure described above, air is always sucked before being injected. Tests differ for the volume accumulated after the suction phase and for the flow rate. Two different syringes have been used depending on the programmed flow rate. For the 3 and 5 mL/min experiments, a syringe with 12.6 mm internal diameter was used, whereas the 1 and 0.1 mL/min tests were performed with a 4.66 mm diameter syringe. 

**Figure 10:**
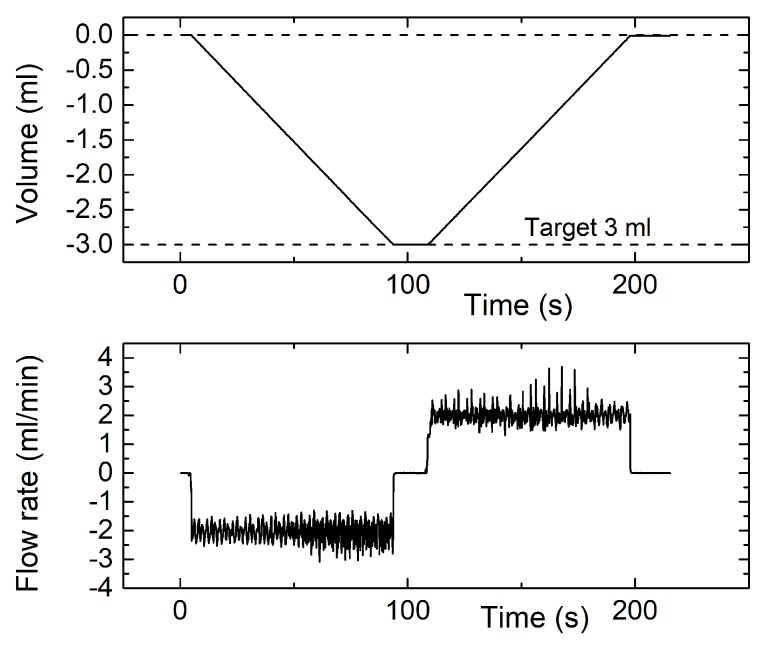
Instantaneous measured volume (**top**) and flow rate (**bottom**) for a measurement cycle obtained by programming the syringe pump for a 3 mL volume and 2 mL/min flow rate.

Figure 10 shows the accumulated volume and instantaneous flow rate during a measurement cycle performed by programming the syringe pump for 3 mL volume at 2 mL/min flow rate. The sharp spikes and noise superimposed to the flow plot are due to the discrete steps applied by the stepping motor that actuates the syringe pump. It should also be observed that the rubber seal of the syringe piston also contributes to the flow fluctuations since, due to friction, the seal edge tends to flutter during the piston motion. Note that, in spite of the mentioned sharp flow changes, volume counting is correct in both the suction and injection phase. The error on the final volume is nearly 0.5%, while the residual volume at the end of the injection phase is only 10 μL. 

**Figure 11:**
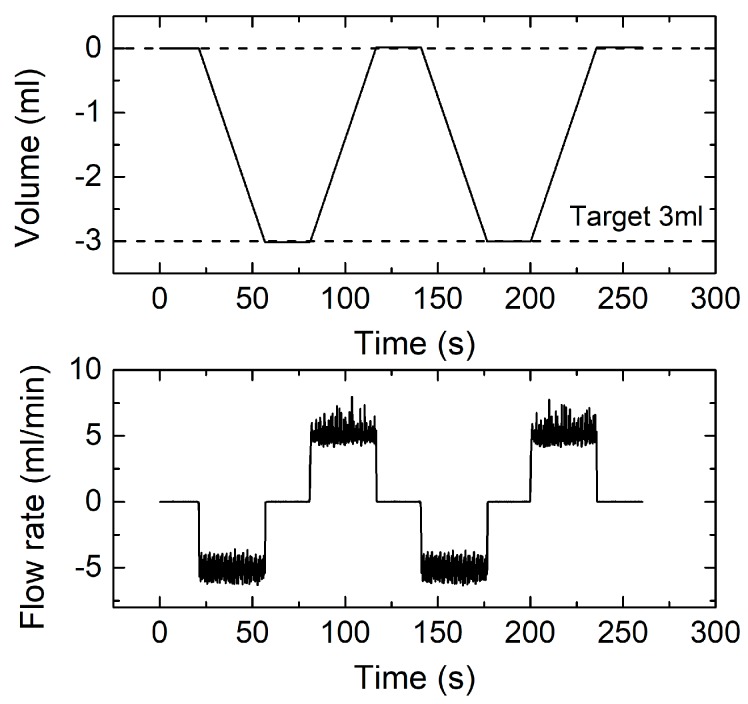
Instantaneous measured volume (**top**) and flow rate (**bottom**) for two successive measurement cycles obtained for 3 mL volume and 5 mL/min flow rate.

Figure 11 shows the results of a test performed programming the same final volume as in Figure 10, but with a 5 mL/min flow. Two successive cycles have been repeated in order to keep the total time similar to the previous case. Again, the target volume is detected with high precision (<0.5%), while the residual volume at the end of the test is just slightly larger (15 μL).

**Figure 12:**
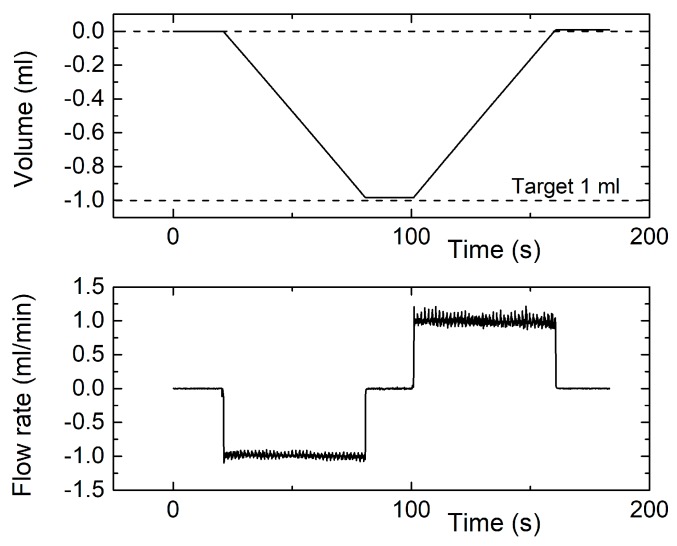
Instantaneous measured volume (**top**) and flow rate (**bottom**) for a measurement cycle obtained by programming the syringe pump for a 1 mL volume and 1 mL/min flow rate.

**Figure 13:**
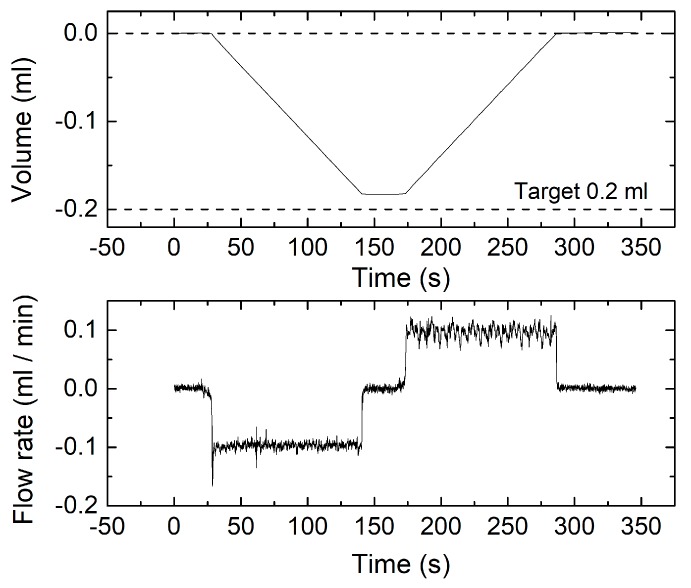
Instantaneous measured volume (**top**) and flow rate (**bottom**) for a measurement cycle obtained by programming the syringe pump for a 0.2 mL volume and 0.1 mL/min flow rate.

Figure 12 shows the result of a volumetric experiment performed with a 1 mL/min rate. The total volume has been reduced to 1 ml, to comply with the use of a syringe of smaller capacity. In this test, the error is significantly higher, since the measured volume was 2% smaller than the programmed one. The error grows up to 8.6% in the test shown in Figure 13, regarding a volumetric experiment performed with a 0.1 mL/min flow rate and 0.2 mL volume. In spite of the lower accuracy, the residual volume is a small fraction of the total one in both tests.

## 4. Conclusions

The results of tests performed at different flow rates show that the proposed sensor can be effectively used to measure gaseous volumes that transit trough a small sized duct. The presence of an irregular flow rate, characterized by short pulses, did not alter the accuracy of the measurements. This property, together with the capability of distinguishing the direction of gas flows, makes this type of sensors suitable for precise metering of gas volumes injected by peristatic or diaphragm pumps. Measurements performed in zero flow conditions across a period of several hours demonstrate the excellent time stability of the offset calibration approach. A residual offset drift, equivalent to nearly 200 nL/(min × °C) has been estimated from the measurements. The observed drift apparently derives from the sensor physics and not from the amplifier. However, considering a conservative full-scale range of 10 mL/min, the drift is only 10 ppm/°C, which demonstrates the effectiveness of the original offset compensation technique. The lower accuracy found at 0.1 mL/min could be ascribed to the fact that such a flow rate practically coincides with the precision of the mass controllers used to calibrate the sensor response.

## Figures and Tables

**Table 1:** Amplifier input differential voltages as a function of the connection-matrix control code.

Code	Amplifier Input Voltage
0	*V_T1_*
1	0 (input short circuited)
2	*V_T2_*
3	*V_T1_*–*V_T2_*
